# Transfer RNA-derived small RNAs (tsRNAs) in gastric cancer

**DOI:** 10.3389/fonc.2023.1184615

**Published:** 2023-07-12

**Authors:** Lu Gan, Haojun Song, Xiaoyun Ding

**Affiliations:** ^1^ Health Science Center, Ningbo University, Ningbo, China; ^2^ The Gastroenterology Department, The First Affiliated Hospital of Ningbo University, Ningbo, China; ^3^ The Biobank of The First Affiliated Hospital of Ningbo University, Ningbo, China

**Keywords:** tsRNAs, tRFs, tiRNAs, gastric cancer, biomarker

## Abstract

Transfer RNA-derived small RNAs (tsRNAs) are newly discovered noncoding RNAs (ncRNAs). According to the specific cleavage of nucleases at different sites of tRNAs, the produced tsRNAs are divided into tRNA-derived stress-inducible RNAs (tiRNAs) and tRNA-derived fragments (tRFs). tRFs and tiRNAs have essential biological functions, such as mRNA stability regulation, translation regulation and epigenetic regulation, and play significant roles in the occurrence and development of various tumors. Although the roles of tsRNAs in some tumors have been intensively studied, their roles in gastric cancer are still rarely reported. In this review, we focus on recent advances in the generation and classification of tsRNAs, their biological functions, and their roles in gastric cancer. Sixteen articles investigating dysregulated tsRNAs in gastric cancer are summarized. The roles of 17 tsRNAs are summarized, of which 9 were upregulated and 8 were downregulated compared with controls. Aberrant regulation of tsRNAs was closely related to the main clinicopathological factors of gastric cancer, such as lymph node metastasis, Tumor-Node-Metastasis (TNM) stage, tumor size, and vascular invasion. tsRNAs participate in the progression of gastric cancer by regulating the PTEN/PI3K/AKT, MAPK, Wnt, and p53 signaling pathways. The available literature suggests the potential of using tsRNAs as clinical biomarkers for gastric cancer diagnosis and prognosis and as therapeutic targets for gastric cancer treatment.

## 1 Introduction

Gastric cancer (GC), the fourth most common cause of tumor-associated death, poses a severe threat to human health worldwide, especially in East Asia (1). The initial symptoms of gastric cancer are not specific or obvious, so most patients have already reached an advanced stage at the time of diagnosis and thus miss the optimal treatment period. Therefore, finding biomarkers that can identify early GC and new targets for GC treatment is of great significance.

Transfer RNA-derived small RNAs (tsRNAs) are newly discovered noncoding RNAs (ncRNAs) that were once mistakenly considered random degradation products of transfer RNAs (tRNAs). In recent years, with the development of high-throughput sequencing technology and advances in bioinformatics analysis, tsRNAs have received increasing attention in cancer research (2, 3). Based on the specific cleavage at different sites of precursor tRNAs or mature tRNAs, the generated tsRNAs are classified into tRNA-derived stress-induced RNAs (tiRNAs) and tRNA-derived fragments (tRFs). Based on the splicing site within the tRNA, tiRNAs can be classified into 5′-tiRNAs and 3′-tiRNAs. tRFs can be classified into tRF-1, tRF-2, tRF-3, tRF-5, and i-tRF. Studies have shown that tRFs and tiRNAs have essential biological functions, such as RNA silencing, translation regulation and epigenetic regulation, and play significant roles in the occurrence and development of various tumors (4, 5). Although the roles of tsRNAs in some tumors have been intensively studied, their roles in GC are still rarely reported.

In this review, we focus on the recent advances in the generation and classification of tsRNAs, their biological functions, and their roles in gastric cancer. We also discuss the potential of using tsRNAs as clinical biomarkers for cancer diagnosis and prognosis and as therapeutic targets for cancer treatment.

## 2 Biogenesis and discovery of tsRNAs

### 2.1 Classification of tRFs and tiRNAs

tRNAs, which have a secondary cloverleaf structure containing three hairpin loops: a D loop, an anti-codon loop, and a TψC loop, play important roles in protein biosynthesis. In recent years, precursor tRNAs, also called mature tRNAs, have been shown to be specifically cleaved to produce a new class of ncRNAs, namely, tsRNAs, which can be divided into tiRNAs and tRFs according to their cleavage sites (5). With the application and development of RNA high-throughput sequencing and analysis, more and more tsRNAs have been discovered (6). The high-throughput sequencing approaches work well for more types of RNAs, such as messenger RNA (mRNA), long ncRNA, microRNA (miRNA), or fragments derived from ribosomal RNA, small nuclear RNA, and small nucleolar RNA (7). Recently, a novel tsRNA- and ribosomal RNA-derived small RNAs (rsRNAs) - friendly small ncRNA sequencing method named PANDORA-Seq (panoramic RNA display by overcoming RNA modification aborted sequencing) has been established, which expands our knowledge on the biogenesis and functions of tsRNAs and rsRNAs, as well as the regulatory roles of various RNA modifications (8).

### 2.1.1 tiRNAs

**Figure 1:**
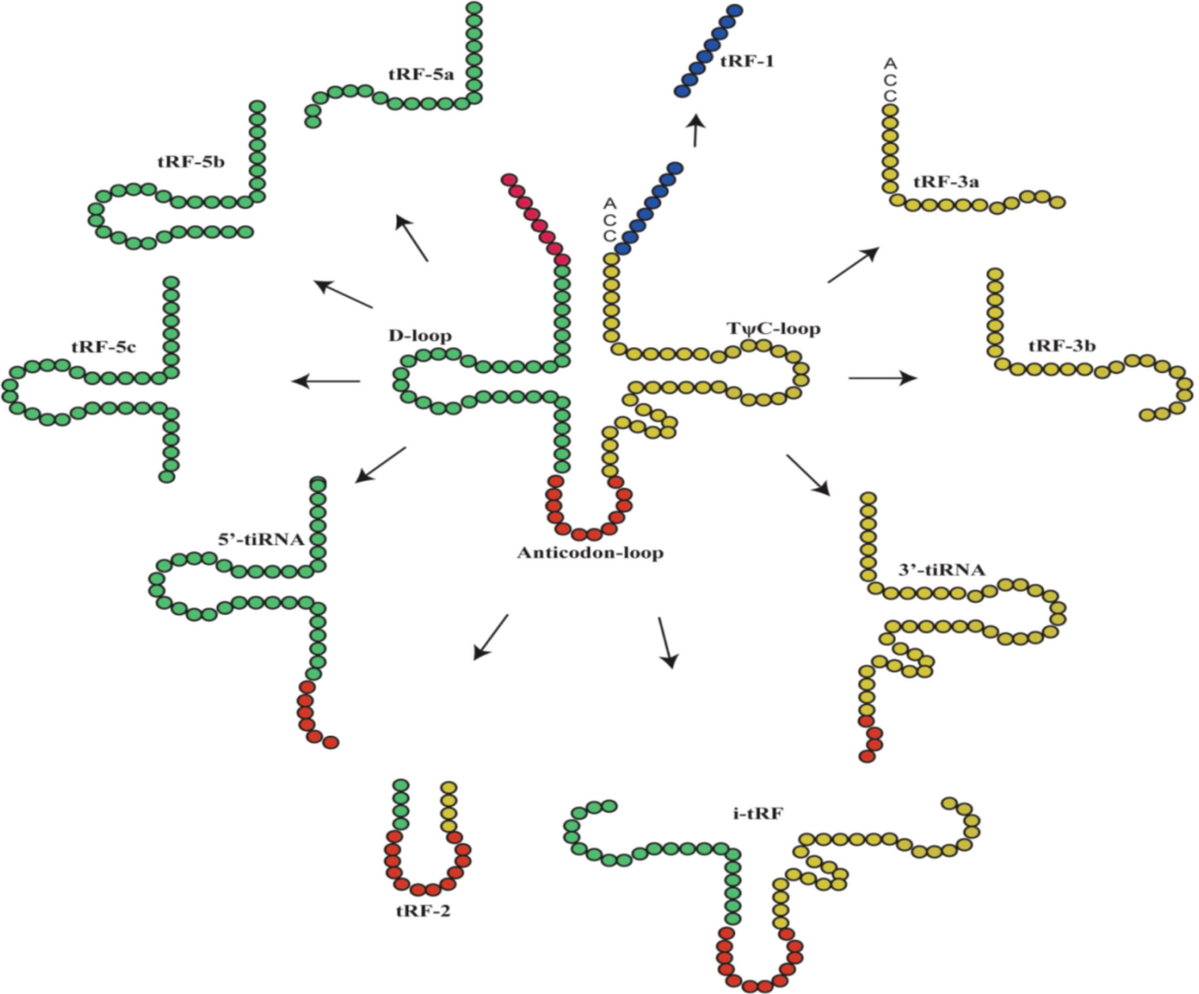
The main classification of tRNA-derived fragments (tRFs) and tRNA halves (tiRNAs). In the figure, the tRNA sources of tRFs and tiRNAs are shown, with the color of the tRNA source matching that of the ncRNA product (9).

tiRNAs, with lengths of 31-40 nucleotides (nt), are generated by cleaving of the anticodon loop of the mature tRNA by angiogenin (ANG) under stress conditions, particularly nutritional deficiency, hypoxia, heat shock, and oxidative stress (9–11). Therefore, tiRNAs are also called tRNA-derived stress-inducible RNAs and tRNA halves. Based on whether the 5′ or 3′ sequencer of the anticodon cleavage position is included, tiRNAs are classified into two basic types: 5′-tiRNAs (including the 5′ end of the mature tRNA to the terminus of the anticodon loop) and 3′-tiRNAs (including the anticodon loop to the 3′ end of the mature tRNA) (**Figure 1**) (9, 11). According to a recent study, it was found that ANG is not the only ribonuclease to produce tiRNAs because the production of some tiRNAs is dependent on RNase L cleavage (12). In addition, the other type of tRNA halves called sex hormone-dependent tRNA-derived RNAs (SHOT-RNAs), which are not induced by various stress stimuli but rather by hormones and are cleaved by ANG, can be highly expressed in hormone receptor-positive breast and prostate cancer cells (13).

#### 2.1.2 tRFs

tRFs have a similar length to miRNAs, approximately 14-30 nt, and can be classified into different types based on their original site in these molecules: tRF-1, tRF-2, tRF-3, tRF-5 and i-tRF (**Figure 1**) (4). tRF-1s are small fragments derived from the 3′ tails of pre-tRNAs and are cleaved by RNase Z or ELAC2 (14, 15). tRF-2s are derived from the decomposition of anticodon loops of tRNAs (6, 15). tRF-3s are derived from the T-loop of the 3′-ends of mature tRNA and are cleaved by ANG and Dicer; they can be further divided into tRF-3a (18 nt) and tRF-3b (22 nt) molecules (4, 14). tRF-5s are produced by Dicer cutting the D loop of tRNA; they can be further divided into tRF-5a (14 nt-16 nt), tRF-5b (22 nt-24 nt) and tRF-5c (28 nt-30 nt) (4, 14). i-tRFs are derived from the anticodon loop of mature tRNAs and sections of the D-loop and T-loop (3, 14, 15).

### 2.2 Nomenclature and databases of tsRNAs

To date, the naming rules of tsRNAs are not consistent, and tRFs are currently more intensively studied than tiRNAs. To promote research and scholarly communication, scientists have created various databases of tsRNAs (**Table 1**), including tRFdb, tDRmapper, tRF2Cancer, MINTmap, MINTbase v2.0, PtRFdb, tRex, tRFexplorer, OncotRF, tsRBase, tRFtarget, and tsRFun (16–27). Additionally, a novel optimized bioinformatic tool, SPORTS1.0 (https://github.com/junchaoshi/sports1.0) can annotate and profile tsRNAs from small RNA (sRNA) sequencing data across a wide range of species and predict potential RNA modification sites based on nucleotide mismatches within sRNAs (28).

**Table 1:** tsRNA databases.

Time	Database	Description	URL link	Reference
**2015**	tRFdb	The first tRFs database containing sequences and read counts of the three classes of tRFs for eight species	http://genome.bioch.virginia.edu/trfdb/	(16)
**2015**	tDRmapper	A standardized nomenclature and quantification scheme are provided to help discover novel tRNA-derived RNA biology	https://github.com/sararselitsky/tDRmapper	(17)
**2016**	tRF2Cancer	A database that allows users to study tRFs expression in different types of cancer	http://rna.sysu.edu.cn/tRFfinder/	(18)
**2017**	MINTmap	A database for rapid and detailed analysis of nuclear and mitochondrial tRNA fragments from short RNA-seq data	https://github.com/TJU-CMC-Org/MINTmap/	(19)
**2018**	MINTbase v2.0	A database including nuclear and mitochondrial fragments from all the cancer genome atlas projects	http://cm.jefferson.edu/MINTbase/	(20)
**2018**	PtRFdb	A database that identifies a total of 5607 unique tRFs in plant	http://www.nipgr.res.in/PtRFdb/	(21)
**2018**	tRex	A Web Portal for Exploration of tRNA-Derived Fragments in arabidopsis thaliana	http://combio.pl/trex	(22)
**2019**	tRFexplorer	A database showing the expression profile of tRFs in every cell line in NCI-60 and for each TCGA tumor type	https://trfexplorer.cloud/	(23)
**2020**	OncotRF	A database for researchers studying the roles, functions and mechanisms of tRFs in human cancers	http://bioinformatics.zju.edu.cn/OncotRF	(24)
**2021**	tsRBase	A database for expression and function of 121942 tsRNAs in multiple species	http://www.tsrbase.org	(25)
**2021**	tRFtarget	A database covering 936 tRFs and 135 thousand predicted targets in eight species	http://trftarget.net	(26)
**2022**	tsRFun	A database for decoding human tsRNA expression, functions and prognostic value across 32 types of cancers	http://rna.sysu.edu.cn/tsRFun/or http://biomed.nscc-gz.cn/DB/tsRFun/	(27)

Currently, there are differences in the nomenclature of tsRNAs in different databases and studies (16, 20, 29, 30). In the tRFdb database, tRFs of each organism are named in the order in which they were identified, with the first tRF-5 named 5001, the first tRF-3 named 3001, and the first tRF-1 named 1001. Also, the tRF-5s with lengths of 15, 22 and 31 nt are attached with “a”, “b” or “c”, respectively, and the tRF-3s with lengths of 18 and 22 nt are attached with “a” and “b”, respectively (e.g., tRF-3019a, tRF-3017A) (16). The TDRmapper database provides a name for each tsRNA containing three components that indicate the parent tRNA “family” from which it is derived, the size, and the region in the mature or pre-tRNA from which it is derived (17). MINTbase v2.0 derives a unique license plate name for each tRF based on the sequence (e.g., tRF-19-3L7L73JD) (20). In addition, in some studies, tRFs are also named by their length (e.g., tRF-25) (31, 32).

In conclusion, scholars have continued to study databases on tsRNAs in recent years, discovering an increasing number of tsRNAs and exploring their mechanisms of action. However, establishing a standardized nomenclature rule for tiRNAs and tRFs has faced several problems, including the extensive chemical modifications of tRNAs, difficulty in obtaining the exact origin of tsRNAs, and the fact that only a few tsRNAs have been experimentally validated (30).

In order to facilitate understanding and memory, a new and consistent naming method based on the structure and sequence of tsRNAs will be adopted in this paper. Taking “tRF -+1: T18 Arg ACG-3-M1-17: C>A” as an example, the name can be divided into 5 parts: tRF, +1: T18, Arg ACG-3, M1, 17: C>A. The first component of the name indicates the types of tsRNAs, which can be divided into tRFs and tiRNAs, and “tsRNA” means those not included in the databases. The second component of the name indicates the region in the mature or pre-tRNA from which the read is derived, which includes three situations that “1:18”, “+1:T18” and “-1:L18”, representing tsRNAs alignment to the 1:18 base of mature tRNA, to the 1:18 base of pre-tRNA 3’tailer, and to the 1:18 base of pre-tRNA 5’leader, respectively. The third part of the name, “Arg ACG-3”, where “Arg” is short for the tRNA amino acid, “ACG” is the anti-codon and “3” is a unique identifier for each tRNA family, represents the tRNA from which tsRNA is derived. The fourth component of the name, “M1”, refers to the comparison of the tsRNA to several tRNAs. If the tsRNA is compared to three tRNAs, it is written as “M3”. The last component of the name is “17:C> A”, where “17” indicates the mutation of the base after base 17 on the tsRNA (i.e., base 18) and “C> A” means that the corresponding base on the tRNA is “C”, while the corresponding base on the tsRNA is “A”.

## 3 Biological functions of tsRNAs

Studies have shown that tsRNAs have a wide range of biological functions, including regulating the stability of mRNA, translation regulation, and epigenetic regulation (2–4, 15). The biological functions of tsRNAs are very complex and require further elucidation. The biological functions and molecular mechanisms studied more thoroughly in recent years are summarized as follows.

### 3.1 Regulating the stability of mRNA

tsRNAs can regulate the stability of mRNAs. On the one hand, some tsRNAs exhibit the same sequences as miRNAs with similar mechanisms of action (15). Huang et al. found that tRF derived from tRNALeu was comparable in sequence to miR-1280 derived from pre-miRNA and inhibited the Notch signaling pathway by directly interacting with the JAG2 mRNA 3′ untranslated region (UTR), thereby inhibiting the proliferation of colorectal cancer cells (33). Some tsRNAs form an RNA-induced silencing complex (RISC) with Argonaute (AGO) proteins in a manner similar to how miRNAs silence mRNA, suggesting that tsRNAs may play a major role in RNA silencing (34). On the other hand, tRFs can bind to RNA-binding proteins (RBPs) and posttranscriptionally regulate gene expression, and RBPs interact with targeted RNAs to control their stability (15). YBX1 is an RBP that is highly overexpressed in several types of cancers. Goodarzi et al. found that under the induction of hypoxic stress in breast cancer cells, some tRFs derived from tRNAAsp, tRNAGlu, tRNAGly and tRNATyr can suppress the stability of multiple oncogenic transcripts by displacing their 3’ UTR from the RBP YBX1 and eventually inhibit the proliferation of breast cancer cells (35).

### 3.2 Translation regulation

tsRNAs can regulate translation levels. A recent study found that LeuCAG3’tsRNA enhances translation by promoting ribosome biogenesis (36). Keam et al. also proposed that the 5’ tRF Gln19 interacts with the human multisynthetase complex (MSC) and increases ribosomal and poly(A)-binding protein translation (37). On the other hand, tsRNAs can inhibit translation by interfering with translation initiation and elongation. Lyons et al. found that G4-tiRNA disrupts the assembly of the 40S ribosomal subunit by directly targeting the HEAT1 structural domain of eIF4G, a major scaffolding protein necessary for translation initiation, ultimately inhibiting translation initiation (38). In addition, Gebetsberger et al. showed that a tRF-5 derived from a valine tRNA-derived fragment (Val-tRF) under certain stress conditions in the halophilic archaeon Haloferax volcanii competes with mRNA for binding to the small ribosomal subunit, thus affecting translation initiation and inhibiting subsequent protein biosynthesis (39).

### 3.3 Epigenetic regulation

Some studies have shown that tsRNAs can act as epigenetic regulators to maintain genome stability by targeting and inhibiting transposable elements (TEs) (40). TEs are DNA sequences that can “move” from one location in the genome to another, and the movement of TEs driven by intact and active transposons is highly mutagenic and must be tightly controlled (41). Therefore, the transcription of TEs is often repressed by epigenetic marks, such as histone modification and DNA methylation (41). Studies have shown that some tRF-3s of 18 and 22 nt in length derived from mature mouse tRNAs could match endogenous retroviruses (ERVs) of long terminal repeats (LTRs), and 22 nt tRFs posttranscriptionally silenced coding-competent ERVs, while 18 nt tRFs specifically interfered with reverse transcription and retrotransposon mobility (42, 43). In addition, Chen et al. revealed that injecting tsRNA fractions from the sperm of male mice fed a high-fat diet into normal zygotes led to metabolic disorders in F1 offspring and altered the gene expression of metabolic pathways in the early embryos and islets of F1 offspring, which was unrelated to DNA methylation at CpG enrichment regions, indicating that sperm tsRNAs represent a paternal epigenetic factor that may mediate the intergenerational inheritance of diet-induced metabolic disorders (44). Zhang et al. revealed that deletion of a mouse tRNA methyltransferase, DNMT2, altered the sperm small RNA expression profile, including levels of tsRNAs and rsRNAs, and abolished sperm small ncRNA-mediated transmission of high-fat-diet-induced metabolic disorders to offspring (45). Recently, Boskovic et al. discovered a specific tRF-5, tRF-Gly-GCC, which plays a role in the production of a variety of ncRNAs. The regulation of U7 snRNA by tRF-Gly-GCC modulates heterochromatin-mediated transcriptional repression of MERVL elements by supporting the production of a sufficient amount of histone proteins (46).

## 4 Roles of tsRNAs in gastric cancer

Growing evidence indicates that tsRNAs play essential roles in tumor development and progression, and their dysregulated expression has some clinical value. In this review, using PubMed, we identified 16 articles published between 2015 and 2022 on dysregulated tsRNAs in GC, involving a total of 17 tsRNAs, of which 9 were upregulated and 8 were downregulated. The potential value of tsRNAs in diagnosing and treating GC is now comprehensively described according to their potential clinical value and the degree of research on their mechanisms. Furthermore, their underlying mechanisms of action in GC were analyzed in depth (**Tables 2**, **3**).

**Table 2:** The clinical value of upregulated tsRNAs in GC and the underlying mechanism.

tsRNA	Unified naming of this paper	Source	Length	Argonaute binding capacity	Sample size	Sample type	Potential clinical value	Observations and correlation with clinical outcome	Putative function and animal experiments	Possible mechanism	Reference
tRF-31-U5YKFN8DYDZDD	tsRNA-32:62-chrM.tRNA2-ValTAC	tRNA-Val-TAC	31nt	Unclear	111	Tissue Serum	Biomarker	High expression is related to late-stage, deep tumor invasion, lymph node metastasis, and vascular invasion.	Not clear yet.	Not clear yet.	(47)
hsa_tsr016141	tRF-1:29-Gln-TTG-1-M3	tRNA-Gln-TTG	29nt	Unclear	193	Tissue Serum	Biomarker	Its levels are linked to differentiation grade, T stage, lymph node status and TNM stage.	Not clear yet.	Not clear yet.	(48)
tRF-23-Q99P9P9NDD	tRF-1:23-Val-CAC-2	tRNA-VAL-CAC	23nt	Unclear	164	Tissue Serum	Biomarker	High expression is positively correlated with T stage, lymph node metastasis, TNM stage and neurological/vascular invasion.	Not clear yet.	Not clear yet.	(49)
tRF-25-R9ODMJ6B26	tRF-52:76-Val-AAC-1-M3	tRNA-Val-AAC	25nt	Unclear	50	Plasma (exosome)	Biomarker	They’re help to identify and diagnose GC patients with a high risk of recurrence and poor prognosis.	Not clear yet.	Not clear yet.	(31)
tRF-38-QB1MK8YUBS68BFD2	tsRNA-39:74-tRNA-Val-AAC-1-M3	tRNA-Val-AAC	38nt	Unclear							
tRF-18-BS68BFD2	tRF-59:76-Val-AAC-1-M5	tRNA-Val-AAC	18nt	Unclear							
tRF-3019a	tRF-58:75-Ala-AGC-1	tRNA-Ala-AGC	18nt	Have proved	112	Tissue	Biomarker	High expression is correlated with TNM stage, histological grade and lymph node metastasis.	High levels promote GC cell proliferation, migration and invasion.	It interacts with the AGO2 protein to silence the tumor suppressor gene FBXO47, thus promoting GC progression.	(50)
							GC promoter				
tRF-3017A	tRF-58:76-Val-TAC-1-M2	tRNA-Val-TAC	19nt	Have proved	87	Tissue	GC promoter	High expression is correlated with positive lymph node metastasis.	High levels promote the invasion and migration of GC cells.	It interacts with the AGO2 protein to silence the tumor suppressor gene NELL2, thus promoting GC progression.	(51)
tRF-60:76-Val-CAC-2	tRF-60:76-Val-CAC-2	tRNA-Val-CAC	17nt	Unclear	65	Tissue	GC promoter	High expression is positively correlated with tumor size and tumor invasion.	High levels promote proliferation and invasion and inhibits apoptosis in GC cells.	It binds to the chaperone molecule EEF1A1, mediates its transport into the nucleus and promotes its interaction with MDM2, thus inhibiting the downstream molecular pathway of p53 and promoting GC progression.	(52)

GC, gastric cancer; AGO2, Argonaute 2; TNM, Tumor-Node-Metastasis.

**Table 3:** The clinical value of downregulated tsRNAs in GC and the underlying mechanism.

tsRNA	Unified naming of this paper	Source	Length	Argonaute binding capacity	Sample size	Sample type	Potential clinical value	Observations and correlation with clinical outcome	Putative function and animal experiments	Possible mechanism	Reference
tiRNA-5034-GluTTC-2	tiRNA-50:84-Glu-TTC-2	tRNA-Glu-TTC	34nt	Unclear	86	Tissue Plasma	Biomarker	Low expression is related to larger tumor size, advanced TNM stage, positive lymphatic metastasis and poor OS.	Not clear yet.	Not clear yet.	(53)
tRF-5026a	tsRNA-1:18-tRNA-Val-AAC-1-M8	tRNA-Val-AAC	18nt	Unclear	86	Tissue Plasma	Biomarker	Low expression is associated with shorter OS, larger tumor size, advanced TNM stage, and positive lymph node metastasis.	High levels inhibite proliferation and migratory capabilities and result in a G0/G1 block. High concentrations of tsRNA-1:18-tRNA-Val-AAC-1-M8 can inhibit tumor growth in mice.	It’s negatively regulated by the PTEN/PI3K/AKT signaling pathway to inhibit GC progression.	(54)
							GC inhibitor				
tRF-24-V29K9UV3IU	tRF-1:24-chrM.Gln-TTG	tRNA-Gln-TTG	24nt	Have proved	19 and 38^*^	Tissue	Biomarker	Not clear yet.	High levels inhibite proliferation, migration, and invasion of GC Cells while promoting apoptosis. Low levels significantly promote tumor growth capacity in mice.	It interacts with the AGO2 protein to silence GPR78, thus inhibiting GC progression. It downregulates the expression of CCND2, FZD3 and VANGL1 to participate in the Wnt signaling pathway and inhibit GC progression.	(55, 56)
							GC inhibitor				
tiRNA-Val-CAC-001	tiRNA-1:34-Val-CAC-2	tRNA-Val-CAC	34nt	Have proved	62	Tissue	Biomarker	Low expression is significantly correlated with advanced TNM stage.	High levels inhibite metastasis, proliferation, and promotes apoptosis of GC cells.	It interacts with the AGO2 protein and then silences LRP6 to regulate the Wnt signaling pathway and inhibit GC progression.	(57)
							GC inhibitor				
tRF-Glu-TTC-027	tRF-+1:T17-Glu-TTC-2-2	tRNA-Glu-TTC	17nt	Unclear	33	Tissue	GC inhibitor	Low expression is related to larger tumor size and advanced histological grade.	High levels suppress the migration and invasion capacities of GC cells. It can significantly reduce the capacity of tumor growth in mice.	It downregulates the expression of ERK1/2, JNK and p38 to inhibit the MAPK signaling pathway and inhibit GC progression.	(58)
tRF-Val-CAC-016	tRF-+1:T15-Val-CAC-1	tRNA-Val-CAC	15nt	Have proved	40	Tissue	GC inhibitor	Its levels are related to tumor size and histology.	It can inhibit the proliferation of GC cells and xenograft tumors in mice.	It interacts with the AGO2 protein and then silences the oncogenic gene CACNA1d to regulate the MAPK signaling pathway and inhibit GC progression.	(59)
tRF-19-3L7L73JD	tsRNA-5:23-tRNA-Val-AAC-1-M7	tRNA-Val-AAC	19nt	Unclear	129	Plasma	Biomarker	Low expression is associated with larger tumor size.	High levels inhibite proliferation and migratory capabilities, increase apoptosis rate and arrest GC cells at the G0/G1 phase.	Not clear yet.	(60)
							GC inhibitor				
tRF-33-P4R8YP9LON4VDP	tiRNA-1:33-Gly-GCC-1	tRNA-Gly-GCC	33nt	Unclear	89	Blood	GC inhibitor	Not clear yet.	High levels arrest GC cells at the G0/G1 phase and inhibit proliferation and migration, promote apoptosis of GC cells.	Not clear yet.	(61)

GC, gastric cancer; AGO2, Argonaute 2; OS, overall survival; TNM, Tumor-Node-Metastasis.

^*^ Two different studies involved 19 and 38 samples, respectively.

### 4.1 The roles of tsRNAs as biomarkers for cancer diagnosis and prognosis

The tsRNAs reported in the literature, whether upregulated or downregulated, have potential clinical value.

#### 4.1.1 Abnormally high expression of tsRNAs as biomarkers in GC

Huang et al. discovered that tsRNA-32:62-chrM.tRNA2-ValTAC exists stably in GC cells, tissues, and serum (47). The levels of tsRNA-32:62-chrM.tRNA2-ValTAC in serum, tumor tissues, and GC cell lines are considerably higher than those in samples from normal physical examination populations and gastritis patients, para-cancerous tissues, and normal gastric epithelial cells. The study found that the expression of serum tsRNA-32:62-chrM.tRNA2-ValTAC in GC patients decreased significantly after the operation. The survival time of patients with high expression of this tRF was significantly shorter than that of patients with low expression. Its high expression is positively correlated with tumor invasion, vascular invasion, Tumor-Node-Metastasis (TNM) stage, and lymph node metastasis. On the other hand, compared with conventional markers, such as CEA, CA199 and CA724, tsRNA-32:62-chrM.tRNA2-ValTAC has higher sensitivity and specificity in the differentiation and diagnosis of malignant and benign gastric tumors. The combined use of tsRNA-32:62-chrM.tRNA2-ValTAC with CEA, CA199 and CA724 has more diagnostic potency and good clinical application potential (47).

Gu et al. revealed that the serum levels of tRF-1:29-Gln-TTG-1-M3 show a gradient change among GC patients, gastritis patients, and healthy donors (48). The increased tRF-1:29-Gln-TTG-1-M3 levels are positively linked to differentiation grade, T stage, lymph node status, and TNM stage. TRF-1:29-Gln-TTG-1-M3 belongs to tRF-5, which is mainly located in the nucleus, and is continuously secreted in tumor cells with good stability and specificity. Receiver operating characteristic (ROC) analysis showed that the serum expression level of tRF-1:29-Gln-TTG-1-M3 can significantly distinguish GC patients from healthy donors or gastritis patients. The sensitivity of tRF-1:29-Gln-TTG-1-M3, CEA, and CA199 in the joint diagnosis of GC is 90%, while that of tRF-1:29-Gln-TTG-1-M3 and CA724 in the joint diagnosis of GC is 82%, which are both higher than the sensitivity of a single tumor marker. Meanwhile, through the analysis of the postoperative survival curve and the expression level of tRF-1:29-Gln-TTG-1-M3 in GC patients, investigators found that tRF-1:29-Gln-TTG-1-M3 can be monitored dynamically in GC patients after the operation. In addition, there is no significant correlation between Helicobacter pylori infection and the expression level of tRF-1:29-Gln-TTG-1-M3 (48).

Zhang et al. found that the differential expression of tRF-1:23-Val-CAC-2 in the serum of GC patients is higher than that of gastritis patients and healthy donors, which can clearly distinguish GC patients from gastritis patients and healthy people (49). The investigators found that serum tRF-1:23-Val-CAC-2 expression levels are significantly reduced in GC patients after the operation, and low expression of the tRF is associated with a prolonged overall survival (OS). High expression is positively correlated with T stage, lymph node metastasis, TNM stage, and neurological/vascular invasion. ROC analysis showed that tRF-1:23-Val-CAC-2 is more effective for early GC diagnosis than the conventional GC biomarkers CEA, CA199 and CA724, and the combination of the tRF and the conventional biomarkers further improves the diagnostic efficiency (49).

Zhang et al. demonstrated that tRF-58:75-Ala-AGC-1 is significantly upregulated in GC tissues and cell lines, and its high expression is correlated with TNM stage, histological grade, and lymph node metastasis (50). Furthermore, ROC analysis showed that tRF-58:75-Ala-AGC-1 could distinguish GC tissue from nontumor adjacent tissues (NATs) with an area under the ROC curve (AUC) of 0.689. Although the diagnostic potential of tRF-58:75-Ala-AGC-1 seems unsatisfactory, it is still superior to previously reported values for CEA (AUC=0.583) and CA199 (AUC=0.585) (50).

Lin et al. discovered that the expression levels of tRF-25 (tRF-52:76-Val-AAC-1-M3), tRF-18 (tRF-59:76-Val-AAC-1-M5), and tRF-38 (tsRNA-39:74-tRNA-Val-AAC-1-M3) in plasma exosomes of GC patients are considerably higher than those of healthy controls (31). The authors developed a GC diagnostic model containing three tRFs (tRF-52:76-Val-AAC-1-M3, tRF-59:76-Val-AAC-1-M5, and tsRNA-39:74-tRNA-Val-AAC-1-M3) with a mean AUC of 0.815, demonstrating greater sensitivity and specificity for GC diagnosis than the commonly used clinical markers and each of the three tRF biomarkers. The results suggested that plasma exosome tRF-52:76-Val-AAC-1-M3, tRF-59:76-Val-AAC-1-M5, and tsRNA-39:74-tRNA-Val-AAC-1-M3 are biomarkers for GC diagnosis and prognosis and help to identify and diagnose GC patients with a high risk of recurrence and poor prognosis (31, 62).

#### 4.1.2 Abnormally low expression of tsRNAs as biomarkers in GC

Zhu et al. found that the expression of tiRNA-50:84-Glu-TTC-2 is downregulated in GC tissues, plasma and GC cell lines, and its level is closely related to tumor size (53). The AUCs of tiRNA-50:84-Glu-TTC-2 in tissue and plasma were 0.779 and 0.835, respectively. When tissues and plasma were used in combination, tiRNA-50:84-Glu-TTC-2 showed a sensitivity, specificity, and AUC of 84.7%, 92.8%, and 0.915, respectively. The OS of patients with lower expression of tiRNA-50:84-Glu-TTC-2 was considerably lower than that of patients with higher expression. Univariate analysis indicated that TNM stage, lymphatic metastasis, and tiRNA-50:84-Glu-TTC-2 expression in tissues are associated with OS, while multivariate analysis revealed that lymphatic metastasis is a detrimental factor for OS (53).

Zhu et al. found that tsRNA-1:18-tRNA-Val-AAC-1-M8 (tRF-18-79MP9P04) is downregulated in GC tissues, plasma samples and GC cell lines, and its levels are considerably associated with OS, tumor size, TNM stage, and lymph node metastasis (54). Animal experiments showed that high concentrations of tsRNA-1:18-tRNA-Val-AAC-1-M8 can effectively inhibit tumor growth in mice. ROC analysis showed that the combination of tissue and plasma tsRNA-1:18-tRNA-Val-AAC-1-M8 as a GC biomarker significantly improves the diagnostic efficiency with an AUC of 0.908 and a sensitivity and specificity of 94.6% and 81.1%, respectively, indicating that tsRNA-1:18-tRNA-Val-AAC-1-M8 can distinguish GC patients from healthy people (54).

Studies found that the AUCs of tRF-1:24-chrM.Gln-TTG and tiRNA-1:34-Val-CAC-2 to differentiate GC tissues from NATs are 0.871 and 0.748, respectively (56, 57). tRF-1:24-chrM.Gln-TTG has good sensitivity and specificity, and the level of tiRNA-1:34-Val-CAC-2 is correlated with TNM stage (56, 57). Additionally, the expression of tsRNA-5:23-tRNA-Val-AAC-1-M7 is downregulated in the plasma of GC patients, and its expression is correlated with tumor size, with an AUC of 0.623 for GC diagnosis (60).

**Figure 2:**
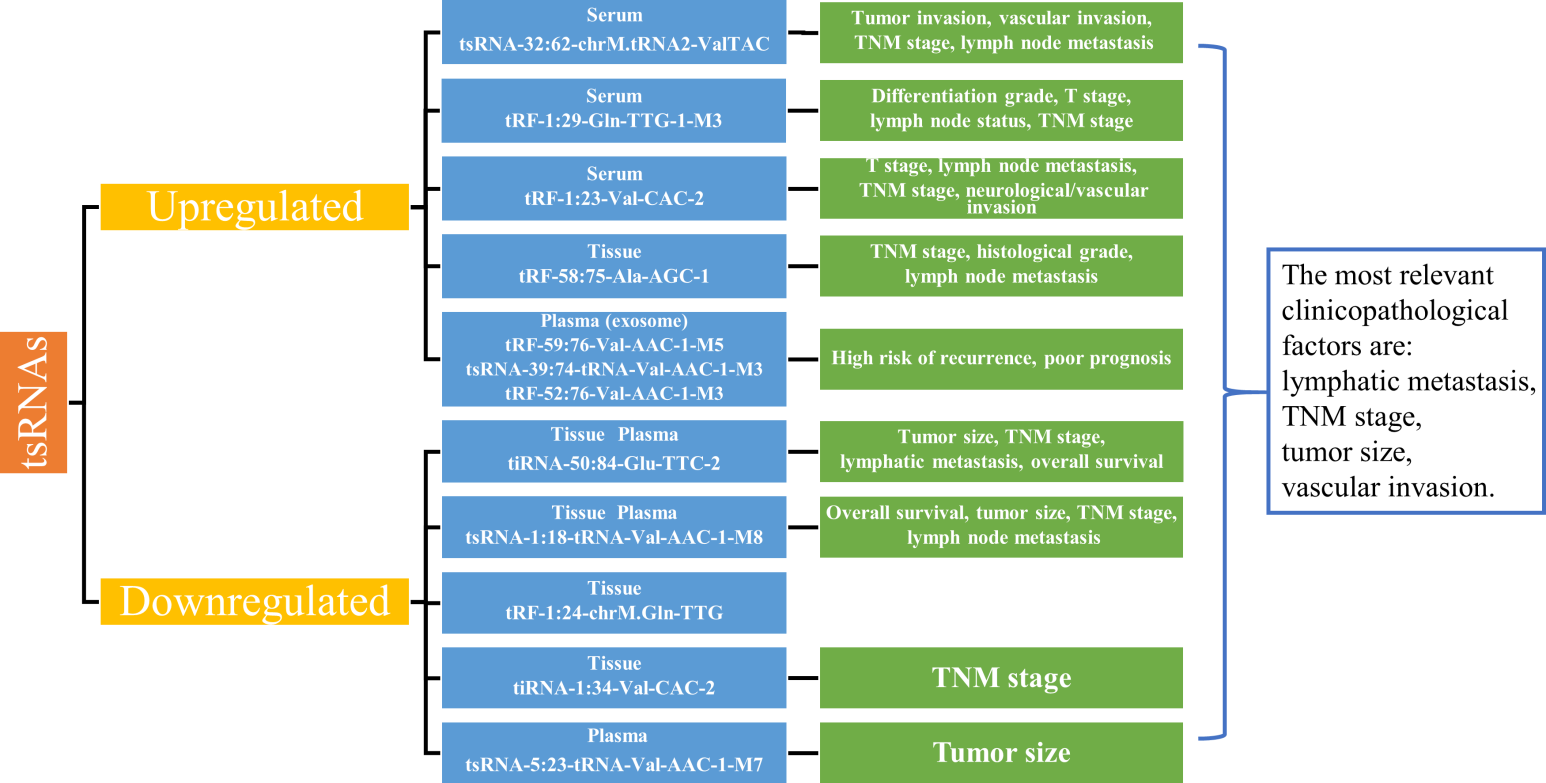
Expression of tsRNAs as biomarkers and correlation with clinicopathological factors. Summarizing the upregulated and downregulated tsRNAs as biomarkers in gastric cancer, the abnormal expression of tsRNAs is closely related to the main clinicopathologic factors of gastric cancer, such as lymph node metastasis, TNM stage, tumor size, and vascular invasion. TNM, Tumor-Node-Metastasis.

tsRNAs are stable and highly enriched in plasma with good sensitivity and specificity. As shown in **Table 4**, tsRNAs have higher diagnostic potency than conventional biomarkers, and tsRNAs in combination with conventional biomarkers show even more significant diagnostic potency. In summary, recent studies found that tsRNA-32:62-chrM.tRNA2-ValTAC, tRF-1:29-Gln-TTG-1-M3, tRF-1:23-Val-CAC-2, tsRNA-39:74-tRNA-Val-AAC-1-M3, tRF-52:76-Val-AAC-1-M3, tRF-59:76-Val-AAC-1-M5 and tRF-58:75-Ala-AGC-1 are significantly upregulated in serum, exosomes and tissues of GC, while tiRNA-50:84-Glu-TTC-2, tsRNA-1:18-tRNA-Val-AAC-1-M8, tRF-1:24-chrM.Gln-TTG, tiRNA-1:34-Val-CAC-2, and tsRNA-5:23-tRNA-Val-AAC-1-M7 are downregulated in tissues and plasma of GC patients (**Table 4**). The differential expression of tsRNAs was closely correlated with the clinicopathological factors of GC, with tsRNAs being highly correlated with lymph node metastasis, TNM stage, tumor size, and vascular invasion (**Figure 2**). In conclusion, tsRNAs could serve as novel biomarkers for GC diagnosis.

**Table 4:** Diagnostic value of tsRNAs as biomarkers in GC.

TsRNA	Sample	Biomarker	AUC	SEN	SPE
tsRNA-32:62-chrM.tRNA2-ValTAC	Serum from GC patients and healthy donors	tsRNA-32:62-chrM.tRNA2-ValTAC	0.740	60.4%	80.9%
		CEA	0.696	57.7%	67.4%
		CA199	0.600	36.0%	82.0%
		CA724	0.639	42.3%	74.2%
		tsRNA-32:62-chrM.tRNA2-ValTAC+CEA	0.783	68.5%	79.8%
		tsRNA-32:62-chrM.tRNA2-ValTAC+CA199	0.769	71.2%	73.0%
		tsRNA-32:62-chrM.tRNA2-ValTAC+CA724	0.771	64.0%	80.9%
		tsRNA-32:62-chrM.tRNA2-ValTAC+CEA+CA199+CA724	0.813	82.0%	69.7%
	Serum from GC patients and gastritis patients	tsRNA-32:62-chrM.tRNA2-ValTAC	0.680	58.6%	72.9%
		CEA	0.651	57.7%	62.5%
		CA199	0.568	36.0%	70.8%
		CA724	0.602	42.3%	68.7%
		tsRNA-32:62-chrM.tRNA2-ValTAC+CEA+CA199+CA724	0.713	86.5%	47.9%
tRF-1:29-Gln-TTG-1-M3	Serum from GC patients and healthy donors	tRF-1:29-Gln-TTG-1-M3	0.814	74.6%	78.2%
		CEA	0.705	68.5%	72.7%
		CA199	0.607	60.0%	63.6%
		tRF-1:29-Gln-TTG-1-M3+CEA	0.830	86.1%	66.4%
		tRF-1:29-Gln-TTG-1-M3+CA199	0.854	–	–
		tRF-1:29-Gln-TTG-1-M3+CEA+CA199	0.864	90.0%	66.4%
	Serum from GC patients and gastritis patients	tRF-1:29-Gln-TTG-1-M3	0.692	63.8%	68.0%
		tRF-1:29-Gln-TTG-1-M3+CEA	0.703	76.9%	60.0%
		tRF-1:29-Gln-TTG-1-M3+CA199	0.683	–	–
		tRF-1:29-Gln-TTG-1-M3+CEA+CA199	0.718	80.8%	52.0%
	Serum from GC patients and healthy donors	tRF-1:29-Gln-TTG-1-M3	0.820	65.6%	89.1%
		CA724	0.780	63.5%	77.0%
		tRF-1:29-Gln-TTG-1-M3+CA724	0.893	82.3%	85.1%
	Serum from GC patients and gastritis patients	tRF-1:29-Gln-TTG-1-M3	0.754	65.6%	80.0%
		CA724	0.716	63.5%	68.6%
		tRF-1:29-Gln-TTG-1-M3+CA724	0.802	87.5%	62.9%
tRF-1:23-Val-CAC-2	Serum from GC patients and healthy donors	tRF-1:23-Val-CAC-2	0.783	66.9%	85.7%
		CEA	0.715	59.7%	73.9%
		CA199	0.614	44.3%	77.3%
		CA724	0.751	53.2%	85.7%
		tRF-1:23-Val-CAC-2+CEA+CA199+CA724	0.862	83.1%	48.7%
	Serum from early GC patients and healthy donors	tRF-1:23-Val-CAC-2	0.724	60.0%	84.9%
		CEA	0.664	51.2%	73.9%
		CA199	0.590	41.5%	77.3%
		CA724	0.711	46.3%	85.7%
		tRF-1:23-Val-CAC-2+CEA+CA199+CA724	0.819	78.0%	47.9%
	Serum from GC patients and gastritis patients	tRF-1:23-Val-CAC-2	0.685	59.7%	74.0%
		CEA	0.678	59.7%	86.0%
		CA199	0.605	44.3%	80.0%
		CA724	0.676	53.2%	84.0%
		tRF-1:23-Val-CAC-2+CEA+CA199+CA724	0.764	81.4%	48.0%
tRF-52:76-Val-AAC-1-M3	Plasma from GC patients and healthy donors	tRF-52:76-Val-AAC-1-M3	0.825	–	–
tsRNA-39:74-tRNA-Val-AAC-1-M3		tsRNA-39:74-tRNA-Val-AAC-1-M3	0.803		
tRF-59:76-Val-AAC-1-M5		tRF-59:76-Val-AAC-1-M5	0.817		
tRF-58:75-Ala-AGC-1	GC tissues and NATs	tRF-58:75-Ala-AGC-1	0.689	–	–
	GC stage I-II tissues and NATs	tRF-58:75-Ala-AGC-1	0.796	–	–
	GC stage III-IV tissues and NATs	tRF-58:75-Ala-AGC-1	0.665	–	–
	GC tissues with lymph node metastasis and NATs	tRF-58:75-Ala-AGC-1	0.754	–	–
	GC tissues without lymph node metastasis and NATs	tRF-58:75-Ala-AGC-1	0.530	–	–
	Poorly differentiated GC tissues and NATs	tRF-58:75-Ala-AGC-1	0.677	–	–
tiRNA-50:84-Glu-TTC-2	GC tissues and NATs	tiRNA-50:84-Glu-TTC-2	0.779	73.3%	46.5%
	Plasma from GC patients and healthy donors	tiRNA-50:84-Glu-TTC-2	0.835	97.3%	59.5%
	GC tissues and NATs, plasma from GC patients and healthy donors	tiRNA-50:84-Glu-TTC-2	0.915	84.7%	92.8%
tsRNA-1:18-tRNA-Val-AAC-1-M8	GC tissues and NATs	tsRNA-1:18-tRNA-Val-AAC-1-M8	0.631	51.2%	72.1%
	Plasma from GC patients and healthy donors	tsRNA-1:18-tRNA-Val-AAC-1-M8	0.883	97.3%	67.6%
	GC tissues and NATs, plasma from GC patients and healthy donors	tsRNA-1:18-tRNA-Val-AAC-1-M8	0.908	94.6%	81.1%
	GC tissues and NATs, plasma from GC patients and healthy donors	tsRNA-1:18-tRNA-Val-AAC-1-M8+tiRNA-50:84-Glu-TTC-2	0.938	91.9%	86.5%
tRF-1:24-chrM.Gln-TTG	GC tissues and NATs	tRF-1:24-chrM.Gln-TTG	0.871	78.6%	92.9%
tiRNA-1:34-Val-CAC-2	GC tissues and NATs	tiRNA-1:34-Val-CAC-2	0.748	–	–
tsRNA-5:23-tRNA-Val-AAC-1-M7	Plasma from GC patients and healthy donors	tsRNA-5:23-tRNA-Val-AAC-1-M7	0.623	40.4%	79.6%

AUC, area under the ROC curve; SEN, sensitivity; SPE, specificity; GC, gastric cancer; NATs, nontumor adjacent tissues.

### 4.2 Mechanisms of tsRNA regulation of GC progression and their potential targets for GC diagnosis and treatment

#### 4.2.1 tsRNAs promote GC progression

Zhang et al. revealed that the upregulated tRF-58:75-Ala-AGC-1 interacts with the AGO2 protein to form a complex and suppress the expression of the target gene *FBXO47* by directly binding to the 3’UTR of the tumor suppressor gene *FBXO47*, which silences the tumor suppressor effect of *FBXO47* and further promotes GC cell proliferation, migration and invasion (50).

Tong et al. found that tRF-58:76-Val-TAC-1-M2 is upregulated in GC tissues and cell lines (51). The upregulated tRF-58:76-Val-TAC-1-M2 forms an RISC with the AGO2 protein, which promotes the migration and invasion of GC cells by directly binding to the 3’UTR of the tumor suppressor gene *NELL2* and silencing the tumor suppressor effect of the *NELL2* gene *via* a mechanism similar to that of miRNA-mediated target gene silencing (51).

Cui et al. showed that tRF-60:76-Val-CAC-2 is significantly upregulated in GC tissues and cell lines (52). tRF-60:76-Val-CAC-2 mediates its translocation into the nucleus by binding to the chaperone molecule EEF1A1, thereby facilitating its interaction with the MDM2-p53 complex and promoting the phosphorylation of MDM2 to enhance the nuclear localization and ubiquitin ligase activity of MDM2. This results in inhibition of the downstream molecular pathway of the tumor suppressor p53, which promotes proliferation and invasion and inhibits apoptosis in GC cells (52, 63).

#### 4.2.2 tsRNAs inhibit GC progression

Zhu et al. discovered that tsRNA-1:18-tRNA-Val-AAC-1-M8 is downregulated in GC cells (54). Increasing tsRNA-1:18-tRNA-Val-AAC-1-M8 levels in both normal and cancerous gastric mucosal epithelial cells results in a G0/G1 block, which arrests the cell cycle and inhibits migration and cell proliferation (54). In addition, tsRNA-1:18-tRNA-Val-AAC-1-M8 is negatively regulated by the PTEN/PI3K/AKT signaling pathway. Low levels of tsRNA-1:18-tRNA-Val-AAC-1-M8 lead to low levels of PTEN as well as elevated levels of PI3K and AKT, which suggests that tsRNA-1:18-tRNA-Val-AAC-1-M8 may exert its tumor suppressor effect through the PTEN/PI3K/AKT signaling pathway (54).

Wang et al. demonstrated that tRF-1:24-chrM.Gln-TTG expression is significantly downregulated in GC tissues, and animal experiments showed that low levels of tRF-1:24-chrM.Gln-TTG significantly promote tumor growth capacity in mice (55). tRF-1:24-chrM.Gln-TTG can function as a miRNA-like fragment, bind to the AGO2 protein, and directly silence the expression of GPR78, a member of the G-protein-coupled receptor superfamily, by complementing the 3’UTR of GPR78 mRNA, thereby inhibiting the proliferation, invasion and metastasis of GC cells and promoting their apoptosis (55). In another study, Dong et al. constructed a network of tRF-1:24-chrM.Gln-TTG target genes involved in cancer development and metastasis, such as ErbB (ErbB2), MAPK (MAP3K7, MAP2K3, MAPK8IP1, MAPK8IP2, MAPK8IP3, etc.), Wnt/β-catenin (WNT4, WNT10A, WNT3A, etc.), and chemokines (CXCR5, CXCR3, CX3CL1, CX3CR1, CXCL9, etc.) by biomimetic analysis (56). The investigators discovered that tRF-1:24-chrM.Gln-TTG further inhibits GC cell proliferation, migration and invasion by downregulating the expression of CCND2, FZD3 and VANGL1 to participate in the Wnt pathway while promoting apoptosis. In addition, tRF-1:24-chrM.Gln-TTG can significantly enrich MAPK. It was speculated that it may participate in the critical process of tumor progression through the p38 MAPK signaling pathway and may also participate in the occurrence and metastasis of GC by targeting organs, lymphatic circulation, Th1/Th2 cell differentiation and inflammation (56).

Zheng et al. found that a 5′-tiRNA named tiRNA-1:34-Val-CAC-2 is significantly downregulated in GC cells and tissues and inhibits GC cell proliferation and metastasis (57). The upregulated tiRNA-1:34-Val-CAC-2 promotes its interaction with the AGO2 protein to silence the expression of LRP6, an essential membrane protein receptor of the Wnt/β-catenin signaling pathway, by targeting LRP6 mRNA in a miRNA-like manner and further inhibits the metastasis and proliferation of GC cells and promotes the apoptosis of GC cells through the Wnt/β-catenin signaling pathway (57).

Xu et al. disclosed that tRF-+1:T17-Glu-TTC-2-2 is significantly downregulated in GC tissues and cells, and animal experiments showed that tRF-+1:T17-Glu-TTC-2-2 could significantly reduce the capacity of tumor growth in mice (58). The upregulated tRF-+1:T17-Glu-TTC-2-2 inhibits the MAPK signaling pathway by downregulating the expression of ERK1/2, JNK and p38, thus significantly inhibiting the proliferation, migration and invasion of GC cells both *in vitro* and *in vivo* (58).

Xu et al. also found that tRF-+1:T15-Val-CAC-1 is significantly expressed at low levels in GC cell lines and tissues, and animal experiments showed that tRF-+1:T15-Val-CAC-1 could inhibit the proliferation of xenograft tumors in mice (59). tRF-+1:T15-Val-CAC-1 regulates the classical MAPK signaling pathway and inhibits the proliferation of GC cells by interacting with the AGO2 protein and then silencing the expression of the oncogenic gene *CACNA1d* (59).

Shen et al. revealed that tsRNA-5:23-tRNA-Val-AAC-1-M7 shows lower expression in GC patient plasma samples and GC cell lines, and the upregulated expression of tsRNA-5:23-tRNA-Val-AAC-1-M7 arrests GC cells at the G0/G1 phase, thereby inhibiting their migration and proliferation and promoting GC cell apoptosis (60).

Shen et al. also found that tiRNA-1:33-Gly-GCC-1 is downregulated in cell lines and plasma of GC patients (61). The upregulated expression of tiRNA-1:33-Gly-GCC-1 inhibits GC cell proliferation by arresting GC cells at the G0/G1 phase, and it also inhibits GC cell migration and promotes apoptosis (61).

**Figure 3:**
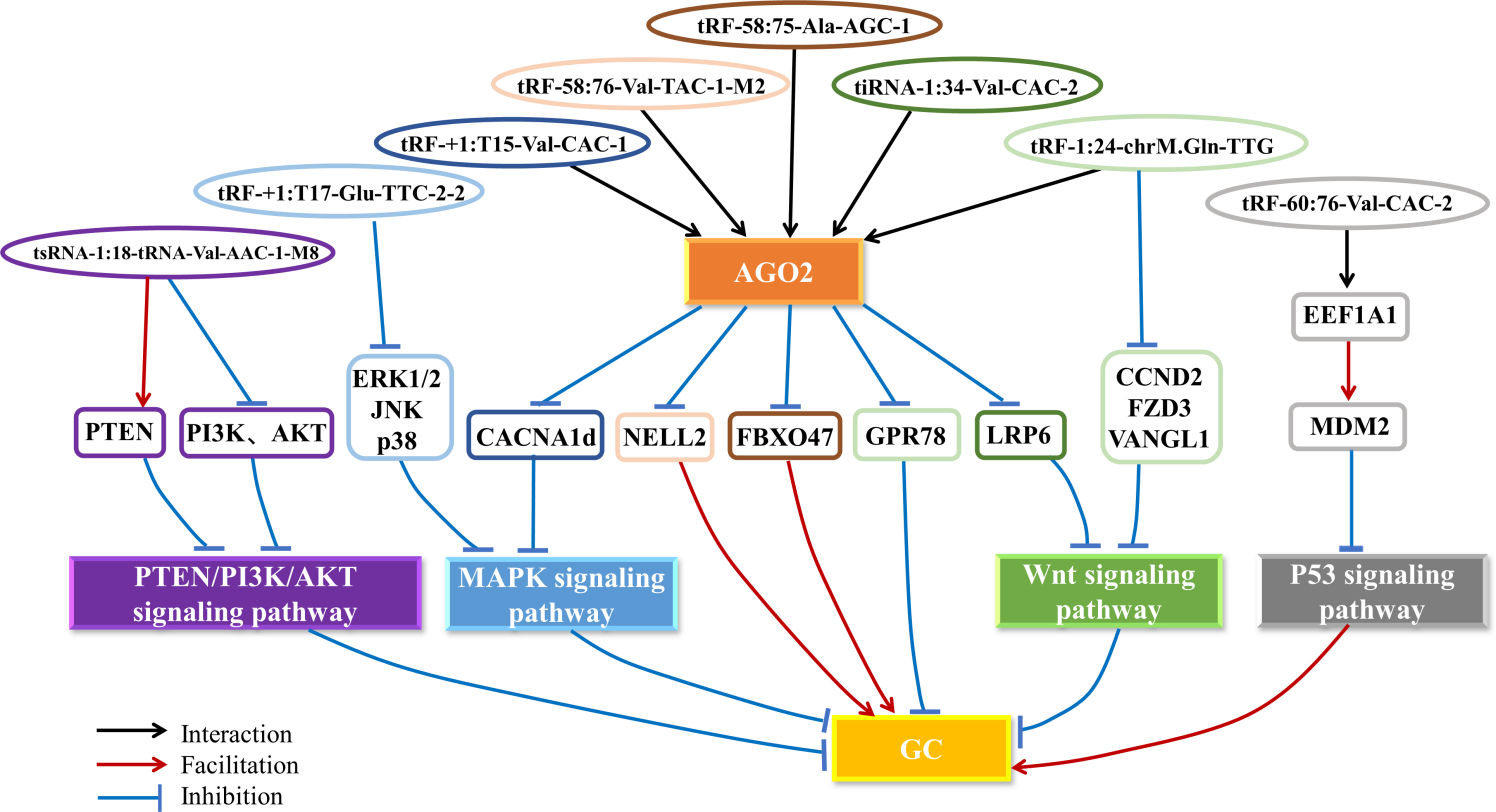
The mechanism of tsRNAs in GC. tsRNAs regulate the PTEN/PI3K/AKT, MAPK, Wnt and p53 signaling pathways by interacting with the AGO2 protein to silence the expression of oncogenes or tumor suppressor genes and other pathways, thus promoting or inhibiting the progression of GC. AGO2, Argonaute 2; GC, gastric cancer.

In summary, tsRNAs play an essential role in tumorigenesis and development (**Figure 3**). We found that tRF-58:75-Ala-AGC-1, tRF-58:76-Val-TAC-1-M2, tRF-+1:T15-Val-CAC-1, tRF-1:24-chrM.Gln-TTG and tiRNA-1:34-Val-CAC-2 have a common feature, that is, they can bind to the AGO2 protein as miRNA-like fragments to silence the expression of target genes and affect the progression of GC (**Figure 3**). Among them, tRF-58:75-Ala-AGC-1 and tRF-58:76-Val-TAC-1-M2 specifically bind to the 3’UTR of *FBXO47* and *NELL2* to form an RISC by interacting with AGO2 and negatively regulate *FBXO47* and *NELL2* expression to promote GC progression, respectively. tRF-+1:T15-Val-CAC-1 and tRF-1:24-chrM.Gln-TTG complement the 3’UTR of *CACNA1d* and GPR78 mRNA, and interact with the AGO2 protein and then silence the expression of *CACNA1d* and GPR78 to inhibit GC progression, respectively. It has been proved that tiRNA-1:34-Val-CAC-2 is regulated by LRP6 by binding to AGO2, and a 3’UTR regulatory site has also been detected, but the correlation between tiRNA-1:34-Val-CAC-2 and 3’UTR of LRP6 has not been explained. Additionally, there is another different mode of action. tRF-60:76-Val-CAC-2 directly binds to the chaperone molecule EEF1A1 and promotes its interaction with MDM2 to inhibit the downstream molecular pathway of p53 and promote GC progression. The researchers only found the effect of tsRNA-5:23-tRNA-Val-AAC-1-M7 and tiRNA-1:33-Gly-GCC-1 on the biological function of gastric cancer and did not explore its regulatory mechanism. Moreover, studies about tsRNA-1:18-tRNA-Val-AAC-1-M8 and tRF-+1:T17-Glu-TTC-2-2 also only discovered the tsRNAs can regulate some important signaling pathways and did not reveal the mechanisms of their action. The other tsRNAs related to GC in our paper were only verified as biomarkers, without in-depth investigation of their mechanisms. There are also some interesting findings that tRF-3a is the main upregulated type of tsRNA in GC, while tRF-5a and tRF-5c are the main downregulated types, suggesting that tRF-3a may play an essential role in the development of GC (52). However, more data is needed to confirm this hypothesis due to the inadequacy of currently available data. These studies suggest that tsRNAs may serve as promising therapeutic targets and biomarkers of GC. Further exploration of tsRNA-related pathways is necessary to establish the GC regulatory network of tsRNAs to help clinicians better diagnose and treat GC.

## 5 Summary and perspectives

With the in-depth exploration of the biological functions and mechanisms of tsRNAs, they are increasingly considered therapeutic targets and new biomarkers for cancer (53). In this report, we review the recent progress in the generation and classification of tsRNAs, their biological functions and their roles in GC and summarize the 17 tsRNAs that have been identified to be upregulated and downregulated in GC, providing evidence to support the clinical value of tsRNAs in GC. However, there are several limitations of the existing studies. First, there are differences in the nomenclature of tsRNAs in different databases and studies, which is an obstacle for summarizing tsRNAs; thus, a standardized nomenclature rule and a high-coverage database are needed for a more concise understanding of tsRNAs. Second, the mechanism of tsRNA dysregulation in GC needs to be studied more deeply, especially in regards to tiRNAs, the data for which are insufficient; the current research is mostly focused on miRNA-like mechanisms of action. Third, the involved signaling pathways only include the Wnt/β-catenin, MAPK, PTEN/PI3K/AKT and p53 signaling pathways. Many studies are needed to confirm the existence of other pathways and fully understand the regulatory network of tsRNAs in GC. Finally, chemotherapy resistance is one of the challenges of GC clinical treatment, and there are no reports of tsRNAs involved in GC resistance mechanisms in the literature. It is still urgent to study whether tsRNAs are involved in drug resistance mechanisms. In conclusion, tsRNAs bring new hope to GC diagnosis and treatment, but further research is needed before they can be put into clinical applications.

## Data Availability Statement

Data sharing is not applicable to this article as no new data were created or analyzed in this study.

## Author contributions

All authors listed have made a substantial, direct, and intellectual contribution to the work and approved it for publication
